# Supplementary material for: Potentiation of cerebellar Purkinje cells facilitates whisker reflex adaptation through increased simple spike activity
Source: eLife. 2018 Dec 18;7:e38852. doi: 10.7554/eLife.38852 (PMC6326726; doi:10.7554/eLife.38852)
Supplement: Supplementary file 1 — (A) Overview of statistical tests on whisker movements – belonging to Figure 1—figure supplement 2. (B) Overview of statistical tests – belonging to Figure 5. (C) Overview of statistical tests – belonging to Figure 8—figure supplement 1. [file elife-38852-supp1.docx]

**Table S1 – Overview of statistical tests on whisker movements – belonging to Figure 1 – figure supplement 2**

| Stimulus condition | Maximal retraction | ***p* < 0.001**; Fr = 18.200; df = 3; *n* = 9 mice  Friedman’s two-way ANOVA | | |
| --- | --- | --- | --- | --- |
|  |  | Front contra | Back ipsi | Front ipsi + PPI |
| Front ipsi | -3.06° (1.19°) | *p* = 0.865 | ***p* < 0.001** | *p* = 0.268 |
| Front contra | -1.76° (0.73°) |  | ***p* = 0.037** | *p =* 1.000 |
| Back ipsi | -0.71° (1.00°) |  |  | *p* = 0.171 |
| Front ipsi + PPI | -1.67° (0.84°) |  |  |  |
|  | Maximal protraction | ***p* = 0.003**; Fr = 13.933; df = 3; *n* = 9 mice  Friedman’s two-way ANOVA | | |
| Front ipsi | 6.10° (5.88°) | ***p* = 0.021** | *p* = 1.000 | *p* = 1.000 |
| Front contra | 12.80° (4.95°) |  | *p* = 0.268 | ***p =* 0.003** |
| Back ipsi | 5.69° (4.29°) |  |  | *p* = 0.865 |
| Front ipsi + PPI | 4.24° (2.04°) |  |  |  |

Summary of statisical analysis belonging to Figure 1 – figure supplement 2. Data are represented as medians (inter-quartile range). Bold values indicate *p* values considered to be statistically significant. contra = contralateral; df = degrees of freedom; ipsi = ipsilateral; PPI = paired-pulse inhibition.

**Table S2 – Overview of statistical tests – belonging to Figure 5.**

| Whiskers | Maximal protraction |  | |
| --- | --- | --- | --- |
| Pre-induction | 9.72° (6.51°) | ***p* < 0.001** | Wilcoxon matched-pairs test |
| Post-induction | 11.39° (9.10°) | *n* = 16 mice |  |
| Complex spikes | Maximal response | | |
| Pre-induction | 1.27% (1.89%) | *p* = 0.163 | Wilcoxon matched-pairs test |
| Post-induction | 1.23% (1.22%) | *n* = 55 PCs |  |
| Simple spikes | Spike rate (norm.) | | |
| Pre-induction | 98.50% (27.3%) | ***p* = 0.003** | Wilcoxon matched-pairs test |
| Post-induction | 102.84% (23.70%) | *n* = 55 PCs |  |

Summary of statisical analysis belonging to Figure 5. Data are represented as medians (inter-quartile range). Bold values indicate *p* values considered to be statistically significant.

**Table S3 – Overview of statistical tests – belonging to Figure 8 – figure supplement 1.**

| Basic firing properties (panels B-D) | | | |
| --- | --- | --- | --- |
| Complex spike frequency | | ***p* = 0.014**; df = 2; Kruskal-Wallis test | |
| WT^1^ | 1.33 Hz (0.61 Hz) |  | *n* = 147 PCs |
| L7-PP2B | 1.04 Hz (0.59 Hz) | ***p* = 0.010**^2^ | *n* = 37 PCs |
| L7-GluA3 | 1.12 Hz (0.59 Hz) | *p* = 0.780^2^ | *n* = 25 PCs |
| Simple spike frequency | | ***p* < 0.001**; df = 2; Kruskal-Wallis test | |
| WT^1^ | 75.86 Hz (33.59 Hz) |  | *n* = 147 PCs |
| L7-PP2B | 51.72 Hz (15.67 Hz) | ***p* < 0.001**^2^ | *n* = 37 PCs |
| L7-GluA3 | 84.78 Hz (25.60 Hz) | *p* = 0.289^2^ | *n* = 25 PCs |
| Simple spike CV2 | | ***p* < 0.001**; df = 2; Kruskal-Wallis test | |
| WT^1^ | 0.35 (0.15) |  | *n* = 147 PCs |
| L7-PP2B | 0.25 (0.13) | ***p* < 0.001**^2^ | *n* = 37 PCs |
| L7-GluA3 | 0.43 (0.14) | *p* = 0.252^2^ | *n* = 25 PCs |
| Complex spike response properties (panels E-G) | | | |
| Amplitude (% of spikes / 1 ms bin) | | ***p* = 0.034**; df = 2; Kruskal-Wallis test | |
| WT^1^ | 1.34% (1.97%) |  | *n* = 129 PCs |
| L7-PP2B | 0.96% (1.26%) | ***p* = 0.019**^2^ | *n* = 34 PCs |
| L7-GluA3 | 0.85% (1.08%) | ***p* = 0.007**^2^ | *n* = 19 PCs |
| Latency to peak | | *p* **=** 0.733; df = 2; Kruskal-Wallis test | |
| WT^1^ | 39 (23) ms | *-* | *n* = 129 PCs |
| L7-PP2B | 42 (16) ms | *-* | *n* = 34 PCs |
| L7-GluA3 | 53 (13) ms | *-* | *n* = 19 PCs |
| Simple spike response properties (panels H-J) | | | |
| Amplitude (% of baseline) | | ***p* < 0.001**; df = 2; Kruskal-Wallis test | |
| WT^1^ | 89.47% (89.22%) |  | *n* = 137 PCs |
| L7-PP2B | 31.00% (47.19%) | ***p* < 0.001**^2^ | *n* = 35 PCs |
| L7-GluA3 | 108.22% (73.47%) | *p* = 0.074^2^ | *n* = 25 PCs |
| Latency to peak | | ***p* < 0.001**; df = 2; Kruskal-Wallis test | |
| WT^1^ | 31 (17) ms |  | *n* = 137 PCs |
| L7-PP2B | 47 (21) ms | ***p* < 0.001**^2^ | *n* = 35 PCs |
| L7-GluA3 | 41 (20) ms | *p* = 0.275^2^ | *n* = 25 PCs |

Summary of statisical analysis belonging to Figure 8 – figure supplement 1. Data are represented as medians (inter-quartile range). Bold values indicate *p* values considered to be statistically significant. df = degrees of freedom; PC = Purkinje cell; PPI = paired-pulse inhibition.

^1^For ease of comparison, WT PCs are grouped (including L7-PP2B WT and L7-GluA3 WT PCs).

^2^Compared to littermate controls.
